# Trauma-Induced Phaeohyphomycosis in an Immunocompetent Host on Dupilumab

**DOI:** 10.7759/cureus.50962

**Published:** 2023-12-22

**Authors:** Ishita Aggarwal, David Arps, Edidiong Kaminska

**Affiliations:** 1 Dermatology, University of Illinois College of Medicine, Chicago, USA; 2 Dermatopathology, Consolidated Pathology Consultants S.C., Libertyville, USA; 3 Dermatology, Kaminska Dermatology, Chicago, USA

**Keywords:** fungal diseases, managing atopic dermatitis, immunocompetent adults, dupilumab, phaeohyphomycosis

## Abstract

Phaeohyphomycosis is a rare infection caused by dematiaceous fungi containing melanin in their cell wall. Patients are often immunocompromised, and cases seen in immunocompromised hosts have increased in recent years. Dupilumab, a monoclonal antibody against interleukin-4 (IL-4) and interleukin-13 (IL-13) used for the treatment of atopic dermatitis, has been linked to hypersensitivity reactions resulting in facial redness, and there is growing evidence that dupilumab may increase susceptibility to yeast infections as well. We present a case of trauma-induced cutaneous phaeohyphomycosis in an immunocompetent host on dupilumab. As dupilumab becomes more commonly encountered in practice, this case is meant to explore the potential relationship between dupilumab and predisposition to opportunistic fungal infections.

## Introduction

Phaeohyphomycosis is an infection caused by dermatiaceous fungi that involves the exposed skin and subcutaneous tissue of the limbs and head and occasionally the paranasal sinuses and central nervous system [1,2]. Infection may follow a penetrating injury with plant material. Patients are often immunocompromised, particularly those who develop systemic infections [2]. Here, we describe a case of trauma-induced cutaneous phaeohyphomycosis in an immunocompetent host on dupilumab.

## Case presentation

**Figure 1:**
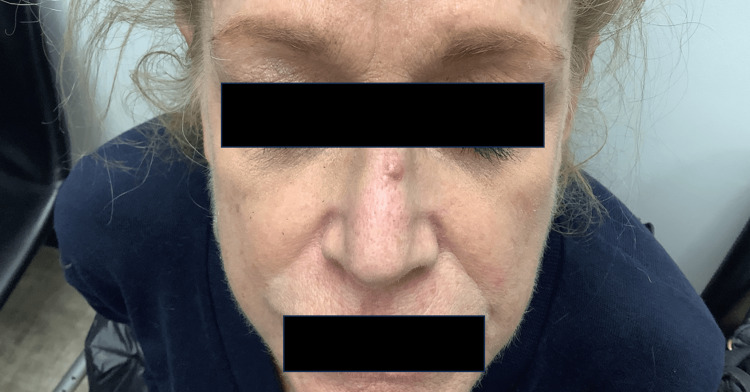
Erythematous nodule on dorsum of nose.

A 66-year-old female with a history of atopic dermatitis, who was recently started on dupilumab, presented with a new erythematous nodule on the dorsum of her nose. She reported that the lesion appeared in the exact area where her nose was injured in a recent biking accident (Figure 1).

**Figure 2:**
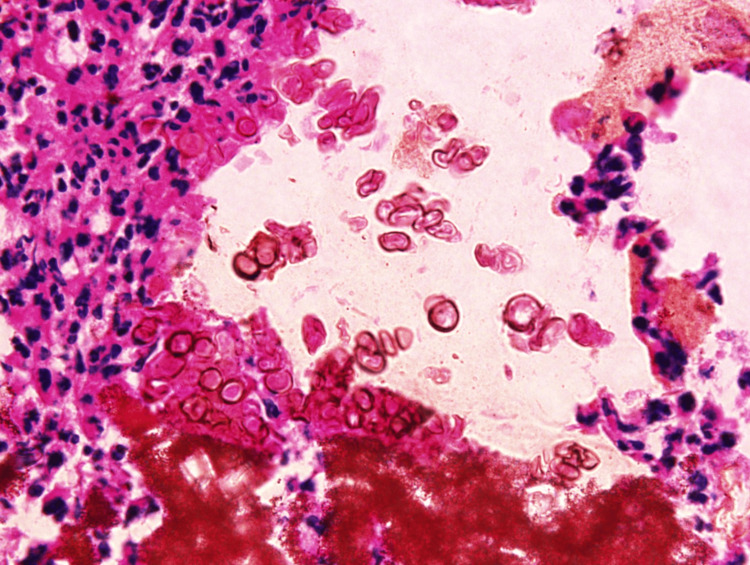
Dense dermal acute inflammation with polarizable foreign material and pigmented fungal organisms (hematoxylin & eosin stain, 100x)

A shave biopsy was performed. Histopathological analysis revealed hypergranulosis, mild spongiosis, and acanthosis within the epidermis. Within the dermis, there were fragments of polarizable foreign material with surrounding dense acute inflammation (Figure 2). 

**Figure 3:**
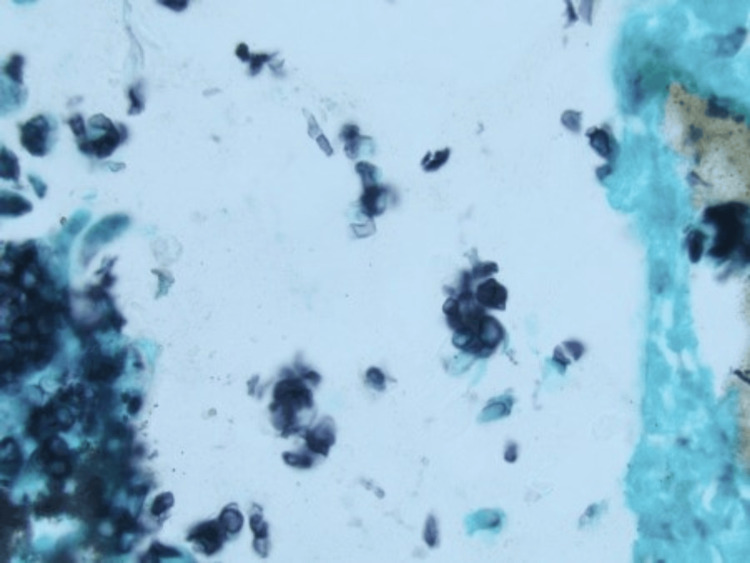
Pigmented fungal organisms, yeast forms, and septate hyphae within the dermis (Gömöri methenamine silver stain, 100x)

Gömöri methenamine silver stain highlighted pigmented yeast forms within the dermis consistent with phaeohyphomycosis (Figure 3). 

The patient was started on oral fluconazole 200 mg weekly for three months and 1% topical econazole twice daily for 30 days, and the infection resolved.

## Discussion

Phaeohyphomycosis is a rare infection caused by a variety of dematiaceous fungi containing melanin in their cell wall [1,3].^ ^While prevalence is unknown, cases seen in immunocompromised hosts have increased in recent years [1].

The disease is classified into cutaneous, subcutaneous, systemic, and cerebral based on the extent and depth of invasion and may present with papulonodules, ulcerated plaques, abscesses, pyogranulomas, and non-healing ulcers [1]. Differential diagnoses include epidermal cysts, bacterial abscesses, foreign body granuloma, and squamous cell carcinoma [1]. Histopathologically, the lesions show brown-walled septate hyphae, yeast, or a combination of both. Diagnosis can be confirmed with periodic acid-Schiff and/or Gömöri methenamine silver stains. Fontana-Masson stain may also be used to confirm the presence of melanin [1]. The pathogenesis of phaeohyphomycosis is usually secondary to direct inoculation from local trauma [1,3]. Melanin in the fungal cell wall scavenges free radicals produced by phagocytic cells, acting as the key virulence factor to make infection in immunocompetent hosts possible [1,4].

There is growing evidence that dupilumab may increase susceptibility to yeast infections. It is theorized that in patients with an already-disrupted skin barrier, opportunistic fungi can more easily penetrate the skin and exacerbate local inflammation [5]. Our patient had a history of atopic dermatitis and was initiated on dupilumab one month prior to developing phaeohyphomycosis. While dupilumab has not been directly linked to phaeohyphomycosis, it has been linked to hypersensitivity resulting in facial redness [6]. Thus, in patients with a history of atopy and dupilumab use, recognizing the role of opportunistic fungi, known for involvement in type I IgE-mediated hypersensitivity reactions, is critical and requires histopathology for specific diagnosis.

Antifungal therapy in combination with surgical excision is recommended for managing local phaeohyphomycosis with itraconazole considered the standard therapy even in cases of incomplete resection or relapse [1,3,4]. Our patient was started on fluconazole after insurance denied itraconazole coverage. Good treatment outcomes are seen in up to 76.2% of patients; poor outcomes often result from delayed or misdiagnosis, ineffective treatment against the specific causative organism, or the occurrence of disseminated systemic infection that is often refractory to therapy [1,3].

## Conclusions

The present case demonstrates trauma-induced phaeohyphomycosis in an immunocompetent individual with pre-existing atopic dermatitis, on dupilumab, and a history of trauma to the nose. This case emphasizes the need to further explore the relationship between dupilumab and potential predisposition to fungal infections as this agent becomes more commonly utilized and the prevalence of phaeohyphomycosis grows, and to utilize histopathology to make a definitive diagnosis, which may prevent long-term scarring and poor outcomes.
